# Crystallized but not soluble uric acid elicits pro-inflammatory response in short-term whole blood cultures from healthy men

**DOI:** 10.1038/s41598-019-46935-w

**Published:** 2019-07-19

**Authors:** Henrik Brovold, Trine Lund, Dmitri Svistounov, Marit D. Solbu, Trond G. Jenssen, Kirsti Ytrehus, Svetlana N. Zykova

**Affiliations:** 1 Metabolic and Renal Research Group, UiT The Arctic University of Norway, Tromsø, Norway; 2 Cardiovascular Research Group, UiT The Arctic University of Norway, Tromsø, Norway; 3 Section of Nephrology, University Hospital of North Norway, Tromsø, Norway; 4 Center for Quality Assurance and Development, University Hospital of North Norway, Tromsø, Norway; 5 Department of Blood Bank and Medical Biochemistry, Innlandet Hospital Trust, Lillehammer, Norway; 6 Department of Transplantation Medicine, Oslo University Hospital and University of Oslo, Oslo, Norway

**Keywords:** Interleukins, Experimental models of disease

## Abstract

Several epidemiological studies have pointed at serum uric acid (SUA) as an independent risk factor for mortality, diabetes, hypertension, cardiovascular and kidney disease; however, no clear pathogenic pathway is established. Uric acid (UA) crystals show pro-inflammatory properties and can thus create or contribute to the state of chronic low-grade inflammation, a widely accepted pathogenic mechanism in several of the above-mentioned pathologies. On the other hand, soluble uric acid possesses antioxidant properties that might attenuate inflammatory responses. We aimed to explore the net effects of experimentally rising SUA in human whole blood cultures on several mediators of inflammation. Production of TNF-α, IL-1ß, IL-1RA, MCP-1 and IL-8 was assessed upon addition of 200 µM UA, 500 µM UA or monosodium urate (MSU) crystals in the presence or absence of 5 ng/ml lipopolysaccharide (LPS). RT-qPCR and multiplex bead based immunoassay were used to measure mRNA expression and cytokine release at 2 and 4 h of culture, respectively. ^14^C labeled UA was used to assess intracellular uptake of UA. We show that crystallized, but not soluble, UA induces production of pro-inflammatory mediators in human whole blood. Soluble UA is internalized in blood cells but does not potentiate or reduce LPS-induced release of cytokines.

## Introduction

Hyperuricemia is a modifiable condition that can lead to gout, and may be an independent risk factor for mortality^1^, renal disease^2,3^, cardiovascular events^4–7^, cancer^8,9^, hypertension^10,11^, and diabetes^12^. Hyperuricemia is also a common problem after renal, liver and cardiac transplantation^13^.

Epidemiological studies are conflicting whether elevated serum uric acid (SUA) is an independent risk factor for cardiovascular disease. Some studies have found SUA to be an independent cardiovascular risk factor^1,4,5,7,14^, but others did not^15–18^.

In humans, the concentration of uric acid (UA) can reach its theoretical supersaturation threshold in extracellular fluids. This is due to multiple missense mutations in the uricase enzyme responsible for degrading UA to a more soluble allantoin in most other species. Precipitation of uric acid into monosodium urate (MSU) crystals in joints elicits acute aseptic inflammation, the gouty arthritis, prevalence of which increases with increasing SUA levels^19^. Deposition of MSU crystals can also occur in other tissues and organs in humans, although it is reported seldom^20–22^. Formation of MSU crystals may also occur in the blood from hyperuricemic patients^22^.

MSU crystals have recently been suggested as a damage associated molecular pattern (DAMP) which stimulates both the innate and adaptive immune system^23–25^. However, the loss of functional uricase and increase in SUA might represent an evolutionary benefit for humans. UA is recognized to be a major antioxidant in human plasma^26^ and some studies have suggested a neuroprotective effect^27–29^. UA´s potential physiological role is further supported by the high rate of reabsorption of filtered UA in the kidneys^25,30^. Soluble UA has been shown to have both antioxidative and pro-oxidative properties in different animal and *in vitro* experimental models^31^. Anti-inflammatory effects have also been reported for other antioxidants added to lipopolysaccharide (LPS)-stimulated whole blood cultures^32,33^. One doctoral thesis reported that UA attenuated inflammatory responses to LPS indirectly by inhibiting the release of pro-inflammatory cytokines TNF-α and IL-1ß in a human monocyte culture, this effect, however, could not be attributed to antioxidant properties of UA^34^. Little is known with respect to the direct effect of elevated SUA on inflammatory markers in blood. Therefore, we aimed to investigate whether short-term exposure of blood cells to high concentration of UA elicit a pro-inflammatory response or compromise cell viability, either directly or through precipitation into MSU crystals. Alternatively, UA might elicit protective effects in blood cells exposed to inflammatory stimuli. We also examined to what extent UA is internalized by blood cells.

## Results

### Soluble UA and blood cell viability

**Figure 1:**
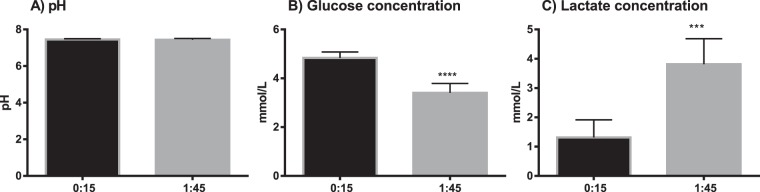
Metabolic parameters in whole blood suspension cultures exposed to vehicle (0.9% NaCl) for 15 minutes (baseline) or for 1 hour 45 minutes (end of culture). The results are paired samples, n = 6 in both groups. The bars and whiskers represent mean + SD (**A**) pH (**B**) Glucose (**C**) Lactate. ***p < 0.001, ****p < 0.0001.

**Figure 2:**
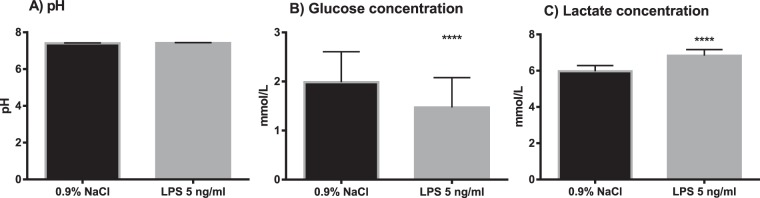
Metabolic parameters in whole blood suspension cultures exposed to vehicle (0.9% NaCl) or lipopolysaccharide (LPS, 5 ng/ml) for 3 h 45 min. The results are paired samples, n = 7 in both groups. The bars and whiskers represents the mean + SD. (**A**) pH (**B**) Glucose concentration (**C**) Lactate concentration. ****p < 0.0001.

**Figure 3:**
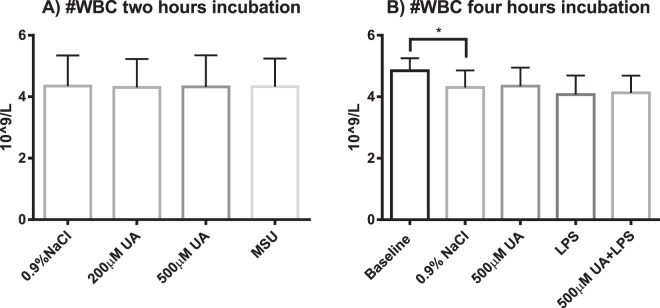
Absolute number of WBC in whole blood suspension cultures subjected to (**A**) 2- hour incubation with vehicle (0.9% NaCl), 200 µM uric acid (UA), 500 µM UA or monosodium urate (MSU) crystals, n = 10; or to (**B**) 4h-incubation with 500 µM UA or vehicle in the presence or absence of 5 ng/ml lipopolysaccharide (LPS) compared to baseline, n = 4. The results are paired samples. The bars and whiskers represents mean + SD. *p ≤ 0.05, **p < 0.001, ****p < 0.0001.

Measurements made in blood cultures at 15 minutes (baseline) and 1 h 45 min of incubation showed that pH did not significantly change during incubation (pH 7.46 ± 0.04 vs. 7.44 ± 0.07 respectively, n = 6, paired t-test, p = 0.3). At the start of incubation, the mean pH in the whole blood cultures was at the upper limit of the reference range of physiological pH for arterial blood (7.35–7.45). As expected, glucose concentration was significantly lower, while lactate concentration was significantly higher at the end of the incubation at 1 h 45 minutes (Fig. 1A–C). There were no significant differences in pH between the cultures exposed to vehicle only and cultures exposed to LPS, after 3 h 45 minutes incubation (pH 7.41 ± 0.02 and pH 7.42 ± 0.03, n = 7, paired t-test, p = 0.2). Vehicle treated cultures had higher glucose concentration at the end of the incubation compared to LPS-treated cells (2.0 ± 0.6 mM vs 1.5 ± 0.6 mM, respectively, n = 7, paired t-test p < 0.0001). Lactate concentration was lower in the vehicle-treated cultures vs LPS (6.0 ± 0.3 mM vs 6.8 ± 0.3 mM respectively, n = 7, paired t-test p < 0.0001) (Fig. 2A–C). Hematological differential cell counts were used as a measure of cell viability in the whole blood cultures. There were no significant differences in the total white blood cell (WBC) count in cultures exposed to 200 µM UA, 500 µM UA or MSU crystals for 2 h compared to vehicle-treated controls (4.3 ± 0.9 10^9^/L, 4.3 ± 1.0 10^9^/L, 4.3 ± 0.9 10^9^/L vs 4.4 ± 1.0 10^9^/L respectively, n = 10) (Fig. 3A). As shown in Fig. 3B, no statistically significant decreases in the total WBC count was observed when whole blood cultures were exposed to 500 µM UA (4.4 ± 0.6 10^9^/L), LPS (4.1 ± 0.6 10^9^/L) or LPS + UA (4.2 ± 0.6 10^9^/L) for 4 h compared to vehicle (4.3 ± 0.6 10^9^/L, adj. p = 0.55, 0.07 and 0.62 respectively, n = 4). When the baseline vehicle sample was compared against vehicle incubated for 4 h, there was a significant decrease in WBC´s (4.9 ± 0.4 10^9^/L vs 4.3 ± 0.6 10^9^/L, p = 0.04, n = 4).

### Cytokine production in whole blood cultures exposed to soluble UA or MSU crystals

**Figure 4:**
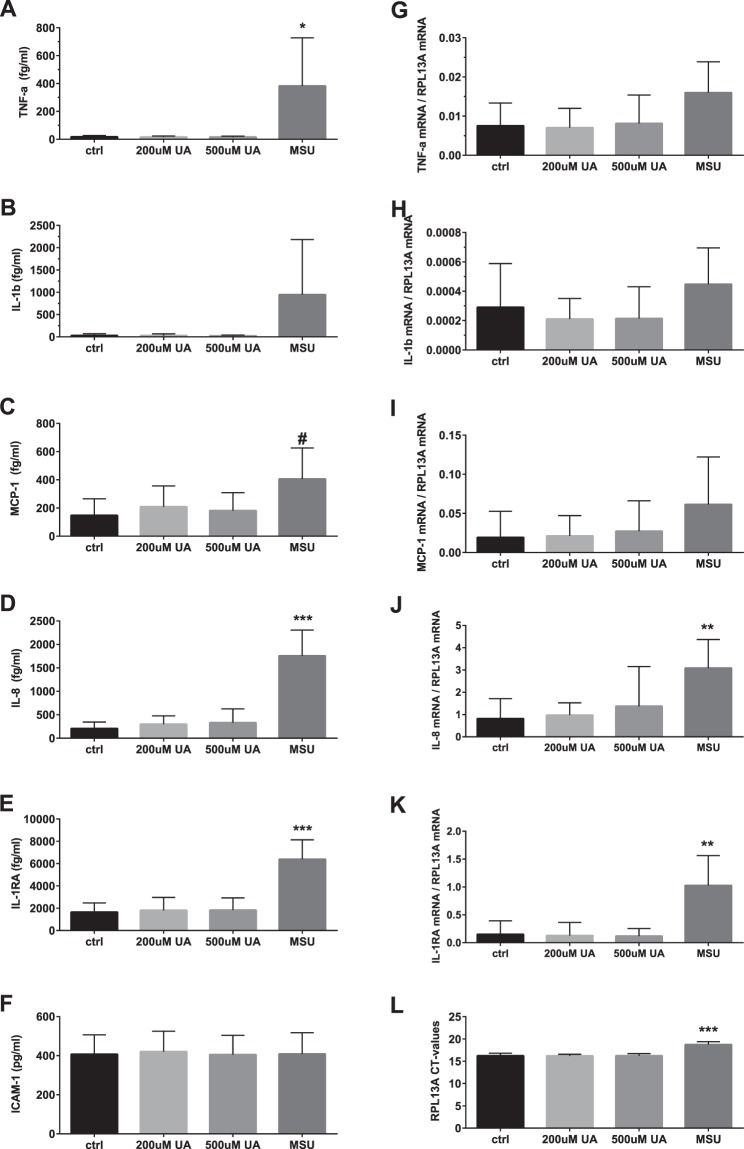
Plasma concentration of TNF-α (**A**), IL-1β (**B**), MCP-1 (**C**), IL-8 (**D**), IL-1RA (**E**), ICAM-1(**F**) and mRNA levels for TNF-α (**G**), IL-1β (**H**), MCP-1 (**I**), IL-8 (**J**), IL-1RA (**K**) normalized to house keeping gene RPL13A (**L**) in whole blood cultures exposed to vehicle (control), 200 µM uric acid (UA), 500 µM UA or monosodium urate (MSU) crystals. The blood was cultured in the presence of uric acid for 3 h for gene expression study and for 5 h for measurement of cytokines. The bars are mean + SD. N = 8 individual donors. ^#^p = 0.055; *p < 0.05; **p < 0.01; ***p < 0.001.

No significant changes in protein concentration of TNF-α, Il-1ß, MCP-1, IL-8 and Il-1RA were observed following exposure of blood cultures to increasing concentrations of soluble UA for 4 h (Fig. 4A–E). At the same time, MSU crystals induced significant release of TNF-α, borderline significant release of MCP-1, and clearly elevated releases of IL-8 and IL-1RA (95% CI of difference from vehicle only exposed controls: 5–724 fg/ml, p adj = 0.047; −7–524 fg/ml, p adj = 0.056; 1008–2092 fg/ml, p adj = 0.0002; 2743–6741 fg/ml, p adj = 0.0005 respectively, n = 8). There were no statistically significant differences between the cultures in IL-1ß concentrations due to wide variation of values. ICAM-1, which expression - as expected - did not change in the course of the experiment, was used as a control.

In order to differentiate between the release of pre-formed cytokines from their *de-novo* production, expression of TNF-α, IL-1ß, MCP-1, IL-8 and Il-1RA mRNA was assessed with qPCR. As illustrated in Fig. 4G–K, addition of 200 µM or 500 µM of soluble UA to whole blood cultures on top of the donors’ natural serum UA level had no significant effect on the cytokines mRNA expression after two hours incubation. At the same time, MSU crystals caused a significant increase in IL-8 and IL-1RA mRNA expression (95% CI of difference from the vehicle: 1.15–3.39, p adj = 0.0014 and 0.38–1.38, p adj = 0.0031 respectively, n = 8). No significant effect of exposure to MSU crystals was observed on TNF-α, IL-1ß and MCP-1 mRNA.

### Effect of soluble UA on LPS-stimulated production of cytokines in whole blood cultures

**Figure 5:**
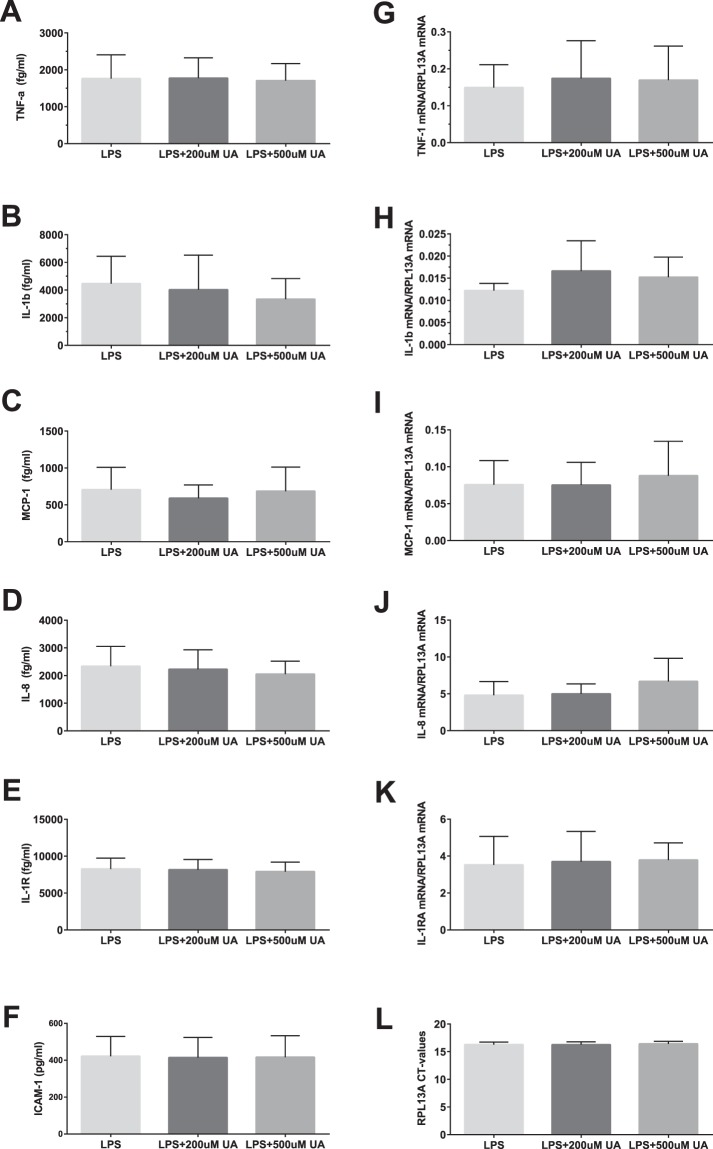
Plasma concentration of TNF-α (**A**), IL-1β (**B**), MCP-1 (**C**), IL-8 (**D**), IL-1RA (**E**), ICAM-1(**F**) and mRNA levels for TNF-α (**G**), IL-1β (**H**), MCP-1 (**I**), IL-8 (**J**), IL-1RA (**K**) normalized to house keeping gene RPL13A (**L**) in whole blood cultures exposed to vehicle (control), 200 µM uric acid (UA) or 500 µM UA and stimulated with LPS (5 ng/ml). Cultures pre-exposed to uric acid for 1 hour were incubated with LPS for 2 h for gene expression study and for 4 hours for measurements of cytokines. The bars are mean + SD. N = 8 individual donors. Cytokine levels were measured in culture plasma with multiplex luminescence immunoassay.

To investigate the potential effect of UA as an antioxidant able to counteract pathogen-induced cytokine and chemokine production, LPS-stimulated production of TNF-α, Il-1ß, MCP-1, IL-8 and Il-1RA was studied in whole blood cultures pre-exposed to vehicle or increasing concentrations of soluble UA for 1 hour *in vitro*. After 4 h of incubation, LPS caused significant release of all the studied cytokines but neither 200 µM or 500 µM UA modulated LPS-responses (Fig. 5A–E). Similarly, LPS-induced increase in the cytokines mRNA expression measured at 2 h was not attenuated by pre-exposure of cultures to either 200 µM or 500 µM UA (Fig. 5G–K).

### Internalization of UA by blood cells

**Figure 6:**
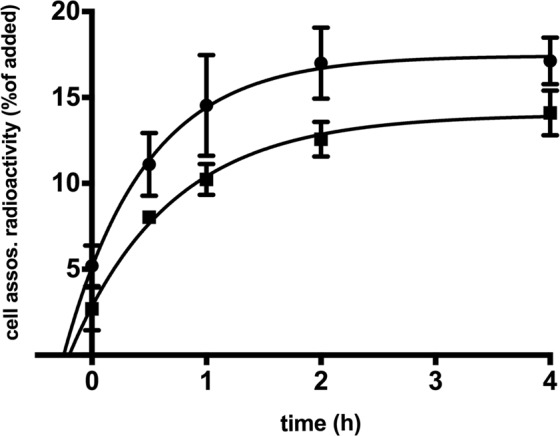
Uptake of ^14^C-labelled UA in whole blood suspension cultures from healthy volunteers. Freshly drawn blood was mixed in equal volumes with RPMI-1640 containing 1000 µM UA and 1 µM ^14^C-labelled UA and incubated at 37 °C 5% CO_2_ for 0, 0.5, 1, 2 and 4 h. Circles – males (n = 3), squares – females (n = 3), data are mean ± SD.

In order to study whether blood cells can internalize UA, whole blood cultures were exposed to high concentration of UA with trace amounts (1‰) of ^14^C-labelled UA, and the radioactivity distribution between plasma and cellular fractions was compared at various time points. The results indicated that cell-associated radioactivity rose rapidly but the uptake process reached equilibrium after 2 h with approximately 17% and 14% of added radioactivity being internalized in male and female groups respectively (Fig. 6). Whereas, overall UA uptake were numerically lower in the female group compared to male (p value 0.0151), the differences in the rate of uptake are minor and not significant (rate constant K 1.409, SE 0.3635 for males and K 1.133, SE 0.1799 for females (p value 0.5002)).

Humans lack the UA-degrading enzyme uricase. However, UA can still be degraded by non-specific oxidation. We did not detect any sign of UA degradation in the blood culture supernatants during 4 hours of incubation (data not shown). The uptake of UA was unaffected by the presence of LPS (5 ng/ml) in the blood cultures, either when added simultaneously or following 1 h of pre-incubation with UA (data now shown). Addition of transporter inhibitors probenecid and tranilast to blood cultures failed to decrease the level of cell-associated radioactivity or the rate of internalization of radiolabeled UA.

## Discussion

Addition of soluble UA to whole blood cultures from healthy male volunteers did not illicit an acute pro-inflammatory response or modulate LPS induced stimulation as judged by unchanged plasma concentration of TNF-α, IL-1ß, IL-1RA, MCP-1 and IL-8. This also applied to UA added in supranormal concentrations. Similarly, the gene expression analysis showed no effect of soluble UA on TNF-α, IL-1ß, IL-1RA, MCP-1 and IL-8 mRNA expression in the cultures. Results from the UA uptake experiment show that UA is internalized by blood cells when added *ex vivo* to whole blood.

Our results are in line with a similar study which showed no pro- or anti-inflammatory effects of supraphysiological concentrations of soluble UA on human whole blood^35^. With regards to the pro-inflammatory properties of soluble uric acid in other species, the reports in the literature are somewhat different. Although not being pro-inflammatory per se, uric acid was convincingly reported to have adjuvant properties as it stimulated expression of co-stimulatory molecules CD86 and CD80 on dendritic cells but did not affect their rate of phagocytosis of particulate antigens. Of note, the concentrations of uric acid that up-regulated co-stimulatory molecules were very close to concentrations at which crystal formation occurs, at least in humans^23^. Another study found that soluble uric acid was able to induce IL-1ß release followed by production of mitochondrial ROS and caspase 1 activation^36^. Both studies used bone-marrow derived *in vitro* maturated macrophages from rodents. Rodents have considerably lower uric acid levels *in vivo* due to presence of functional uricase gene, and the rise in uric acid that macrophages became exposed to *in vitro* (from an assumed baseline of 13 μΜ^37^ to 900 μΜ) was relatively higher than concentration delta in our model (from an assumed baseline of 300 μΜ to 500–800 μΜ). Moreover, in the study by Braga *et al*.^36^ the dose-dependent increase in IL-1ß production in response to soluble uric acid was only observed in the presence of LPS in cultures of rodent macrophages, and no signs of activation were detected in human cells. These results therefore were completely in line with our data.

Addition of soluble UA to LPS-stimulated blood did not have any attenuating effect on the elicited pro-inflammatory response as could be expected from antioxidant properties of UA. Earlier studies in whole blood *ex vivo* have shown that the main chemotactic factors for monocytes and neutrophils, MCP-1 and IL-8 respectively, are regulated by reactive oxygen species (ROS). Adding the exogenous oxygen radical scavengers - N-acetyl cysteine and dimetyl sulfoxide - to LPS-stimulated whole blood, was reported to reduce the release of MCP-1 and IL-8^32,33^. However, a study in monocytes found UA to attenuate the LPS-induced release of cytokines TNF-α and IL-1ß without affecting intracellular ROS, which suggests that the effect might have been mediated by other than antioxidant properties of UA^34^. Due to the natural levels of UA in whole blood from healthy males, isolated monocytes in culture might be a more suitable model to study the potential effect of UA as a molecule with immune-regulatory properties. If UA has immune-regulatory properties, normal SUA levels of healthy individuals might be sufficient for this to occur and there is no additional gain of further increasing UA *ex vivo*.

Nevertheless, addition of crystallized UA to our whole blood cultures caused significant upregulation of mRNA and protein release of several pro-inflammatory cytokines, particularly chemokine IL-8 and IL-1RA. These results contrast a lack of stimulatory effect of soluble UA.

Uric acid is an end product of purine degradation pathway, and an acute rise of serum uric acid in the circulation takes place in situations with massive cell death and break-down of nucleic acids. It is logical that such a sign would be perceived by the immune system as a danger signal. Indeed, high serum uric acid is a predictor of adverse events as it is longitudinally associated with increased mortality and morbidity^1–12^. It is now well established that uric acid crystals are recognized as endogenous danger-associated molecular patterns^23,38,39^. MSU-crystal represents a repetitive purine structure that is likely to be sensed as foreign polynucleotide-like molecule by the cells of immune system. The ability of MSU-crystals to activate pro-inflammatory pathways in phagocytes is linked to engagement of specific receptors^40–44^, membrane rearrangements^45^ and complement activation^46^. Accordingly, lack of sufficient expression or engagement of such receptors as CD14, TLR-2 and −4 and Fc-receptors, for example due to immaturity of phagocytes, insufficient opsonization or blockage of binding sites on MSU-crystals would all prevent an inflammatory response from full activation.

Soluble uric acid as opposed to its crystals is a rather small molecule and its size would prevent it from cross-linking stimulatory receptors on the surface of phagocytes or opsonization. From the evolutionary perspective, it is interesting that in humans, acute elevation of uric acid in its soluble form is not a pro-inflammatory stimulus but crystals are, yet both forms have the same origin and indicate cell death and danger. One possible but speculative explanation is a dialectical transition of quantity into quality where solubility of uric acid indicates that a critical threshold in the number of dead cells is not reached yet, but further rise resulting in formation of crystals, becomes a sign of considerable cellular catastrophe. Crystallization indeed can be augmented by additional danger signals present in necrotic tissues, such as changes in pH, temperature and ion concentrations, or presence of immunoglobulins^47–53^. Indeed, a surprisingly small proportion of people with hyperuricemia experience MSU crystal deposition disease, suggesting either subclinical disease, or that additional factors are needed for MSU crystal formation in human tissue. The conditions in our model with relatively good cell viability (Fig. 3) might therefore not be fully suitable to study the effects of *in vivo* crystallized UA as a DAMP^23,54^.

NLRP3 induced IL-1ß production has been previously implicated as a key element in crystal-elicited pro-inflammatory response^55^. Contrary to this study and several other reports we could not demonstrate significant up-regulation of IL-1ß in response to MSU crystals. We believe this discrepancy is related to the fact that in our model MSU crystals interact with peripheral blood mononuclear cells (PBMC) suspended in plasma and not with adherent maturated macrophages. It has been shown that silica aluminum salt crystals did not induce IL-1β release in human PBMCs unless cells were primed by LPS^56^, a well-known modulator of macrophage differentiation^57^. While studies of mature phagocytes in serum-free environment is relevant as a model for inflammatory response in tissue/joints, whole blood suspension cultures used by us, suit better to study the effects of uric acid in the circulation. As NLPR3 has intracellular localization^58^, its activation with subsequent IL-1ß production requires phagocytic uptake of MSU crystals^56^. Indeed, addition of Cytochalasin D blocks MSU-crystal phagocytosis and abolish activation of NLPR3 inflammasome and IL-1β release^56^. The weak IL-1ß upregulation in our experiments could be attributed to differences in the efficiency of phagocytosis and/or/ density of cell surface receptors between enriched cultures of mature phagocytes and peripheral blood cells. Another possible explanation is the presence of plasma in our cultures which is known to contain elements that can non-specifically antagonize pro-inflammatory responses to crystallized uric acid, such as CD-44^59^ and apolipoprotein B^60^. At the same time, MSU-crystals in our model induced significant increase in the production of other pro-inflammatory cytokines, such as TNF-a and IL-8, confirming that activation of pro-inflammatory pathways has taken place. A more feasible explanation is therefore sub-optimal timing for IL-1ß assessment.

UA has been shown to reduce ROS intracellularly in cultured fibroblast by uptake through GLUT9 (SLC2A)^61^. The results from our UA uptake experiment indicated that UA is indeed internalized into blood cells. However, the type of cells responsible for the uptake remains to be determined. Rough separation of blood suspension 4 h after incubation with ^14^C-UA into erythrocytes and buffy coat fractions showed that the majority of cell-associated radioactivity was present in the erythrocyte fraction (data not shown). Whether white blood cells contribute to ^14^C-UA internalization remains to be determined.

The cell-associated radioactivity detected at the first time point (5.2% and 2.5% for males and females respectively) indicates a very rapid initial uptake of UA. When the uptake experiments were conducted on ice almost no cell-associated radioactivity was detected (1.3%) and no changes in dynamics observed during 4 h (data not shown). This suggests that the uptake of UA by blood cells is not free diffusion across cell membrane the process that would not be significantly hindered by low temperature. Furthermore, the rate of UA uptake suggests that fluid phase pinocytosis is unlikely to be the mechanism behind. Receptor-mediated endocytosis is also unlikely to be involved since no endocytic receptor for UA has been described in humans so far. However, receptor-mediated endocytosis of UA was demonstrated in yeast^62^. Therefore, the most probable mechanism for UA uptake in blood cells is via transmembrane transporters. The possible candidates of UA transporters are GLUT9, URAT1, OAT10 and OAT4 among others^31^. However, addition of inhibitors probenecid and tranilast did not affect uptake of UA (data not shown). Probenecid and tranilast are considered by many to be inhibitors for all the UA transporters mentioned above^63^. However, some authors define probenicid solely as URAT1 inhibitor. Nevertheless, our data indicate that neither GLUT9, URAT1, OAT10 nor OAT4 are involved in UA uptake by blood cells, while it has been previously reported that probenecid retards the uptake of UA, at least in human erythrocytes^64^. It should be noted that with renal tubular epithelial cells as exception, urate uptake mechanisms are not well described. Since only overall UA uptake but not rate of uptake were higher in male group the observed difference in UA uptake between sexes may be due to typically higher erythrocyte numbers in male compared to female subjects.

This study has several limitations. We were not able to measure UA in the cultures because we used anti-coagulated blood and the final concentration of UA in cultures is therefore unknown. The use of only male donors in the experiments studying inflammatory responses weakens the external validity of the study. The number of cytokines and chemokines we measured was limited due to financial restrains. The response to MSU crystals in our study was also somewhat weaker than described by others. This may be due to differences in experimental design, such as longer MSU crystals incubation times or the production of smaller or otherwise qualitatively different crystals in other studies^65^. Based on earlier kinetic experiments performed in similar whole blood models with LPS as stimulant we chose to incubate our cultures for four hours prior to cytokine analysis^32,66^. However, it might be that longer incubation is needed when using MSU crystals as stimulant. No measurements of oxidation products or oxidative burst were conducted in our experiments, and consequently, it is not possible to exclude that UA may have an effect on ROS-production that we did not detect. The timing for sample collection might not be optimal for all the cytokines we wanted to study. Because earlier kinetic experiments performed in similar whole blood models indicated that two hour incubation was probably too short to observe the full effect on MCP-1 and IL-8 release^32,66^, we extended the study with sampling also at four hour time point. Another limitation with the whole blood model for studying chemokines is the presence of Duffy-receptor on red blood cells, which is known to scavenge MCP-1 and IL-8 from plasma^33,67^ and mask the effect of UA on production of these chemokines. At the same time scavenging would doubtfully mask changes in cytokine gene expression, which in our experiments were consistent with ELISA results. In addition, the relatively low number of study participants, the use of young, healthy and mainly male donors are all important limitations.

The strength of this study is the use of freshly drawn human whole blood in an *ex vivo* inflammation model. Exposure of whole blood to high concentrations of UA allows studying the uptake and responses to this metabolite in a setting that is more similar to the situation in the circulation than what can be achieved by using cultures of purified blood cell types or human cell lines. Results from the hematological differential counting and assessment of metabolic parameters indicate that cell viability and metabolism were not considerably affected by incubation of blood for up to four hours *ex vivo*. Humans have higher SUA levels and different purine metabolism than most other mammals, including the typical laboratory animals - rats and mice^30^. The external validity of the study is therefore also superior compared to studies in animal models.

## Conclusion

Crystallized, but not soluble, UA elicits production of pro-inflammatory cytokines TNF-α and IL-1ß, chemokines MCP-1 and Il-8, and IL-RA in short-term cultures of whole blood from healthy young men. Soluble UA was taken up by blood cells but did not seem to potentiate or reduce LPS-induced release of cytokines. The subgroup of blood cell(s) responsible for UA internalization and the mechanism behind need further investigation.

## Methods

### Ethics approval and consent to participate

The study has been approved by the Regional Committee for Medical and Health Research Ethics (2014/73). All the study participants signed the informed consent form prior to participation. The biological material in the study was used without keeping any linked information on the identity or health status of the donors. All methods were performed in accordance with the relevant guidelines and regulations.

### Materials

Uric acid (HPLC quality) (Sigma-Aldrich), Lipopolysaccharide (LPS) from *E*. *coli* 026:B6 (Sigma-Aldrich), EDTA 1% (w/v) in PBS without Ca^2+^ and Mg^2+^ (Versen Biochrome, Germany), RPMI-1640 cell culture medium (Sigma-Aldrich), Tissue solubilizer “Solvable” (PerkinElmer), “Ultima Gold” scintillation cocktail (PerkinElmer), ^14^C labelled uric acid (50 mCi/mmol) (Hartmann Analytic GmbH). Probenecid and tranilast were purchased from Sigma-Aldrich.

### Preparation of solutions

A 4 mM UA stock solution was prepared by dissolving UA powder in 4.06 mM NaOH and pH was adjusted to 8.5 to obtain long-term stable stock solution. The 4 mM UA stock solution was further diluted with 1.8% NaCl to produce 2 mM UA/0.9% NaCl stock solution. All solutions were filtered through a 0.2 µm syringe filter (Acrodisc, Pall corporation, NY, USA) before use. MSU crystals were prepared by addition of NaCl to 4 mM UA stock solution to 0.9%, followed by cooking at 100 °C for 6 h and allowing to precipitate at room temperature under sterile conditions for 5–7 days. The crystal formation was checked microscopically. The crystals were typically 5–25 µm long. LPS was reconstituted in 0.9% NaCl to 500 ng/ml working solution.

### Cytokine production in whole blood suspension culture

A human whole blood suspension model was used as earlier described by others^68^. Healthy male study participants, aged 18–40 years, were recruited from the university, hospital staff members and students. Only males were invited to avoid introducing heterogeneity in the donors from cyclical changes of estrogens during menstrual cycle known to affect immune response and cytokines production^69–71^. Exclusion criteria were self-reported concurrent use of medications or any chronic or acute illness. Blood samples were slowly aspirated from the antecubital vein into a 20 ml polypropylene (BD Falcon) syringe, with a 19-gauge needle and anticoagulated with Dalteparin sodium (Fragmin, Pfizer) 10 IE/ml blood. Aliquots of blood were immediately transferred to 50 ml polypropylene tubes to establish 3 ml blood cultures. UA stock solutions were added to achieve final concentrations of 200 µM or 500 µM on top of the donors’ level to represent moderate and high level of SUA^72^. MSU crystals were added to one of the cultures to a final concentration of 0.78 mg/ml. The volumes of uric acid in solution and crystal suspension added to whole blood cultures, were the same and comprised 1% of total culture volume. The tubes were capped, inverted gently and incubated in a rotary shaker incubator at 37 °C for 1 h, followed by addition of 30 µl of LPS working solution to a final concentration of 5 ng/ml or vehicle. At 2 h the tubes were gently inverted to mix and an aliquot of 2 ml was transferred to a Tempus tube (Applied Biosystems) mixed and kept frozen at −20 °C. The remaining 1 ml of blood cultures continued incubation in a rotary shaker incubator at 37 °C for additional 2 h, followed by centrifugation at 2000g for 10 min. Plasma was then collected and kept frozen at −70 °C until further analyses.

### Quantitative PCR

Total RNA was extracted using the PerfectPure RNA Blood Kit (5′Prime) or RNeasy Mini Kit (Qiagen) according to the manufactures instructions. Isolated RNA was quantified by NanoDrop 1000 Spectrophotometer (Thermo Scientific). Isolated RNA samples were diluted to final concentration 25 ng/µl and cDNA synthesis was conducted with High CapacitycDNA kit (Applied Biosystems) using 500 ng mRNA in a total reaction volume of 20 µL. cDNA synthesis were performed with and without RT enzyme to generate cDNA and RT-free negative controls. Quantitative PCR (qPCR) was performed using gene-specific primers (Table 1). Fast SYBR© Green master mix (Applied Biosystems) or Fast start Essential DNA green Master (Roche), primers and 2 µL of 1:4 diluted cDNA were used in a total of 10 µL reaction volume. cDNA was amplified in duplicates in Roche Light Cycler 96 (Roche). Thermal cycling settings were pre-denaturing at 95 °C for 10 min, followed by 40 cycles of 95 °C for 10 s, 60 °C for 10 s and 72 °C for 10 s. A negative control and RT-free negative control were included in every assay. The following primer pairs were used 5′ to 3′

**Table 1:** Gene-specific primers.

Gene	Forward primer	Reverse primer
TNF-α	TCTTCTCGAACCCCGAGTGA	TAGCCCATGTTGTAGCAAACCCTCAAGCT
MCP-1	TTCTGTGCCTGCTGCTCAT	GGGGCATTGATTGCATCT
IL-8	GTTTTTGAAGAGGGCTGAGAATTC	CATGAAGTGTTGAAGTAGATTTGCTTG
IL-1ß	TGC GAC ACA TGG GAT AAC G	TTT TTG CTG TGA GTC CCG G
IL-1RA	ATACTTGCAAGGACCAAATG	TGTTAACTGCCTCCAGC
RPL-13A	CTGGACCGTCTCAAGGTGTT	GCCCCAGATCAAACTT

The PCR efficiency for all primer sets was determined by performing a serial dilution of pooled cDNA from all individuals. Melting curves were assessed to control for non-specific amplification. RPL-13A was used as a reference gene for normalization of cytokine gene expression (∆C_t_) with adjustment for efficiency of amplification^73^.

### Measurements of cytokines in culture plasma

Luminex® Assay Human Premixed Multi-Analyte Kit (R&D systems) was used for simultaneous measurement of IL-1ß, IL-1RA, TNF-α, IL-8, MCP-1 and ICAM-1 (negative control). The plasma samples were diluted with PBS prior to analysis and analyzed in duplicates according to the manufactures instructions. The chemiluminescense was read on a VERSAmax Absorbance Microplate Reader (Molecular Devices). Final plasma concentrations were calculated using the Bioplex software supplied by the manufacturer. The standard curve was constructed using a 4-parameter logistic function.

### Differential counting and metabolic analyses

Aliquots of blood were pipetted into 15 ml polypropylene centrifugation tubes (BD falcon) containing either vehicle (0.9% NaCl), uric acid (200 µM and 500 µM), MSU crystals (0.78 mg/ml) or LPS (5 ng/ml) to a total volume of 2 ml. The tubes were inverted ten times and incubated with closed caps at 37 °C in a shaker incubator. At baseline and following two or four hour incubation, the experiments were stopped by placing the tubes on ice. A 1000 µl of blood was pipetted to standard EDTA tubes for hematological differential cell counting performed at Clinical Chemistry Department, University Hospital of North Norway. Lactate, glucose, pH, pO_2_, pCO_2_, Na^+^, K^+^, Cl^−^ and Ca^2+^ concentrations were analyzed with an ABL 800 blood gas analyzer (Bergmann diagnostika).

### Measurement of UA uptake by blood cells

Blood from six healthy volunteers (three males and three females) was collected as described above. Blood was mixed 1:1 with RPMI-1640 cell culture medium containing 1 µM ^14^C-UA and 1000 µM non-labelled UA. A total volume of 1790 µl of the mixture was pipetted into six well cell culture plates (BD Falcon) and incubated at 37 °C 5% CO_2_ on a shaker board. Incubation was terminated at 0, 0.5, 1, 2 and 4 h by transferring the cell suspensions to 2 ml microcentrifuge tubes (Eppendorf) and centrifuged at 2000 G for 10 min at 5 °C. Supernatants and cellular fractions were separated and hydrolyzed by incubation with tissue solubilizer “Solvable” at 55 °C for 3 hours according to manufacturers’ instructions. To the hydrolyzed cellular fractions, 50 µl of 0.2 M EDTA and 500 µl of 30% hydrogen peroxide were added and the mixtures were incubated at room temperature for 30 min, and then at 55 °C for 60 min in order to decolorize the samples. After cooling down, both fraction types were mixed with 17.7 ml “Ultima Gold” scintillation cocktail. Radioactivity (d.p.m.) was measured in a Packard Liquid Scintillation Counter. The extent of UA degradation was determined by counting radioactivity after addition to cell culture supernatants of ZnCl_2_ that precipitates only non-degraded UA.

### Inhibition experiments

Incubation on ice was performed in order to differentiate between free diffusion across cell membrane where the dependence on temperature is negligible^74^ and endocytic uptake and facilitated transport (membrane transporters) where effect of temperature lowering is significant^74,75^. Addition of probenecid (final concentration 1 mM) and tranilast (final concentration 100 µM) to blood culture was used to analyze whether GLUT9, URAT1, OAT10 and OAT4 transporters were responsible for uric acid uptake^63^.

### Statistical analysis

Prism 6 and 7 (Graph Pad software inc.) were used to make figures and conduct statistical analyses. A paired t-test was used when two groups were compared with each other. A repeated measures ANOVA with a Geissner-Greenhouse correction for not assuming sphericity was used to compute P values for the main effects in the cytokine experiments. Provided significant differences were observed on an alpha level of ≤ 0.05 in the repeated measures ANOVA, Dunnett’s multiple comparison test was conducted. P-values were adjusted for multiple comparisons. For UA uptake studies, the dynamics of blood cells’ ^14^C-UA-associated radioactivity was analyzed using non-linear regression. The model that best described the radioactivity uptake data, (one phase association model), was selected using the Akaike’s Information Criterion (AIC)^76^. The differences between sex groups were determined by comparing best-fit values of parameters using F-test.

## Data Availability

The data that support the findings of this study are available from UiT - The Arctic university of Tromsø - but restrictions apply to the availability of these data, which were used under license for the current study, and so are not publicly available. Data are, however, available from the authors upon reasonable request and with permission of UiT - The Arctic university of Norway and the Regional committee for medical and health research ethics.

## References

[1] Storhaug HM (2013). Uric acid is a risk factor for ischemic stroke and all-cause mortality in the general population: a gender specific analysis from The Tromso Study. BMC Cardiovasc Disord.

[2] Obermayr RP (2008). Elevated uric acid increases the risk for kidney disease. J Am Soc Nephrol.

[3] Iseki K (2001). Significance of hyperuricemia on the early detection of renal failure in a cohort of screened subjects. Hypertens Res.

[4] Niskanen LK (2004). Uric acid level as a risk factor for cardiovascular and all-cause mortality in middle-aged men: a prospective cohort study. Arch Intern Med.

[5] Fang J, Alderman MH (2000). Serum uric acid and cardiovascular mortality the NHANES I epidemiologic follow-up study, 1971–1992. National Health and Nutrition Examination Survey. JAMA.

[6] Hoieggen A (2004). The impact of serum uric acid on cardiovascular outcomes in the LIFE study. Kidney Int.

[7] Holme I, Aastveit AH, Hammar N, Jungner I, Walldius G (2009). Uric acid and risk of myocardial infarction, stroke and congestive heart failure in 417,734 men and women in the Apolipoprotein MOrtality RISk study (AMORIS). J Intern Med.

[8] Strasak AM (2007). The role of serum uric acid as an antioxidant protecting against cancer: prospective study in more than 28 000 older Austrian women. Ann Oncol.

[9] Strasak AM (2007). Serum uric acid and risk of cancer mortality in a large prospective male cohort. Cancer Causes Control.

[10] Grayson PC, Kim SY, LaValley M, Choi HK (2011). Hyperuricemia and incident hypertension: a systematic review and meta-analysis. Arthritis Care Res (Hoboken).

[11] Sundstrom J (2005). Relations of serum uric acid to longitudinal blood pressure tracking and hypertension incidence. Hypertension.

[12] Bhole V, Choi JW, Kim SW, de Vera M, Choi H (2010). Serum uric acid levels and the risk of type 2 diabetes: a prospective study. Am J Med.

[13] Neal DA, Tom BD, Gimson AE, Gibbs P, Alexander GJ (2001). Hyperuricemia, gout, and renal function after liver transplantation. Transplantation.

[14] Bos MJ, Koudstaal PJ, Hofman A, Witteman JC, Breteler MM (2006). Uric acid is a risk factor for myocardial infarction and stroke: the Rotterdam study. Stroke.

[15] Culleton BF, Larson MG, Kannel WB, Levy D (1999). Serum uric acid and risk for cardiovascular disease and death: the Framingham Heart Study. Ann Intern Med.

[16] Moriarity JT, Folsom AR, Iribarren C, Nieto FJ, Rosamond WD (2000). Serum uric acid and risk of coronary heart disease: Atherosclerosis Risk in Communities (ARIC) Study. Ann Epidemiol.

[17] Wheeler JG, Juzwishin KD, Eiriksdottir G, Gudnason V, Danesh J (2005). Serum uric acid and coronary heart disease in 9,458 incident cases and 155,084 controls: prospective study and meta-analysis. PLoS Med.

[18] Jee SH, Lee SY, Kim MT (2004). Serum uric acid and risk of death from cancer, cardiovascular disease or all causes in men. Eur J Cardiovasc Prev Rehabil.

[19] Richette P, Bardin T (2010). Gout. Lancet.

[20] Ministrini S (2019). Unusual presentation of gouty tophus in the liver with subsequent appearance in the same site of HCC: a correlate diagnosis? Case report. World J Surg Oncol.

[21] Ning TC, Keenan RT (2010). Unusual clinical presentations of gout. Curr Opin Rheumatol.

[22] Martin D (2017). An unusual location of gouty panniculitis: A case report. Medicine (Baltimore).

[23] Shi Y, Evans JE, Rock KL (2003). Molecular identification of a danger signal that alerts the immune system to dying cells. Nature.

[24] Rosin DL, Okusa MD (2011). Dangers within: DAMP responses to damage and cell death in kidney disease. J Am Soc Nephrol.

[25] Rock KL, Kataoka H, Lai JJ (2013). Uric acid as a danger signal in gout and its comorbidities. Nat Rev Rheumatol.

[26] Ames BN, Cathcart R, Schwiers E, Hochstein P (1981). Uric acid provides an antioxidant defense in humans against oxidant- and radical-caused aging and cancer: a hypothesis. Proc Natl Acad Sci USA.

[27] Alvarez-Lario B, Macarron-Vicente J (2011). Is there anything good in uric acid?. QJM.

[28] Chamorro A (2014). Safety and efficacy of uric acid in patients with acute stroke (URICO-ICTUS): a randomised, double-blind phase 2b/3 trial. Lancet Neurol.

[29] Romanos E, Planas AM, Amaro S, Chamorro A (2007). Uric acid reduces brain damage and improves the benefits of rt-PA in a rat model of thromboembolic stroke. J Cereb Blood Flow Metab.

[30] Alvarez-Lario B, Macarron-Vicente J (2010). Uric acid and evolution. Rheumatology (Oxford).

[31] So A, Thorens B (2010). Uric acid transport and disease. J Clin Invest.

[32] Xing L, Remick DG (2007). Mechanisms of oxidant regulation of monocyte chemotactic protein 1 production in human whole blood and isolated mononuclear cells. Shock.

[33] DeForge LE, Fantone JC, Kenney JS, Remick DG (1992). Oxygen radical scavengers selectively inhibit interleukin 8 production in human whole blood. J Clin Invest.

[34] McLaughlin, R. J. *Monocyte Regulation By Soluble Uric Acid* Doctoral Theses thesis, Victoria University of Wellington (2014).

[35] Simon MC (2013). Fatty acids modulate cytokine and chemokine secretion of stimulated human whole blood cultures in diabetes. Clin Exp Immunol.

[36] Braga TT (2017). Soluble Uric Acid Activates the NLRP3 Inflammasome. Sci Rep.

[37] Watanabe T, Tomioka NH, Watanabe S, Tsuchiya M, Hosoyamada M (2014). False *in vitro* and *in vivo* elevations of uric acid levels in mouse blood. Nucleosides Nucleotides Nucleic Acids.

[38] Ghaemi-Oskouie F, Shi Y (2011). The role of uric acid as an endogenous danger signal in immunity and inflammation. Curr Rheumatol Rep.

[39] Kono H, Rock KL (2008). How dying cells alert the immune system to danger. Nat Rev Immunol.

[40] Scott P, Ma H, Viriyakosol S, Terkeltaub R, Liu-Bryan R (2006). Engagement of CD14 mediates the inflammatory potential of monosodium urate crystals. J Immunol.

[41] Liu-Bryan R, Scott P, Sydlaske A, Rose DM, Terkeltaub R (2005). Innate immunity conferred by Toll-like receptors 2 and 4 and myeloid differentiation factor 88 expression is pivotal to monosodium urate monohydrate crystal-induced inflammation. Arthritis Rheum.

[42] Barabe F, Gilbert C, Liao N, Bourgoin SG, Naccache PH (1998). Crystal-induced neutrophil activation VI. Involvment of FcgammaRIIIB (CD16) and CD11b in response to inflammatory microcrystals. FASEB J.

[43] Yagnik DR (2000). Noninflammatory phagocytosis of monosodium urate monohydrate crystals by mouse macrophages. Implications for the control of joint inflammation in gout. Arthritis Rheum.

[44] Naff GB, Byers PH (1973). Complement as a mediator of inflammation in acute gouty arthritis. I. Studies on the reaction between human serum complement and sodium urate crystals. J Lab Clin Med.

[45] Ng G (2008). Receptor-independent, direct membrane binding leads to cell-surface lipid sorting and Syk kinase activation in dendritic cells. Immunity.

[46] Terkeltaub R, Tenner AJ, Kozin F, Ginsberg MH (1983). Plasma protein binding by monosodium urate crystals. Analysis by two-dimensional gel electrophoresis. Arthritis Rheum.

[47] Fiddis RW, Vlachos N, Calvert PD (1983). Studies of urate crystallisation in relation to gout. Ann Rheum Dis.

[48] Iwata H, Nishio S, Yokoyama M, Matsumoto A, Takeuchi M (1989). Solubility of uric acid and supersaturation of monosodium urate: why is uric acid so highly soluble in urine?. J Urol.

[49] Kippen I, Klinenberg JR, Weinberger A, Wilcox WR (1974). Factors affecting urate solubility *in vitro*. Ann Rheum Dis.

[50] Tak HK, Cooper SM, Wilcox WR (1980). Studies on the nucleation of monosodium urate at 37 degrees c. Arthritis Rheum.

[51] Kanevets U, Sharma K, Dresser K, Shi Y (2009). A role of IgM antibodies in monosodium urate crystal formation and associated adjuvanticity. J Immunol.

[52] Kam M, Perl-Treves D, Caspi D, Addadi L (1992). Antibodies against crystals. FASEB J.

[53] Kam M, Perl-Treves D, Sfez R, Addadi L (1994). Specificity in the recognition of crystals by antibodies. J Mol Recognit.

[54] Kono H, Chen CJ, Ontiveros F, Rock KL (2010). Uric acid promotes an acute inflammatory response to sterile cell death in mice. J Clin Invest.

[55] Martinon F, Petrilli V, Mayor A, Tardivel A, Tschopp J (2006). Gout-associated uric acid crystals activate the NALP3 inflammasome. Nature.

[56] Hornung V (2008). Silica crystals and aluminum salts activate the NALP3 inflammasome through phagosomal destabilization. Nat Immunol.

[57] Ziegler-Heitbrock HW, Ulevitch RJ (1993). CD14: cell surface receptor and differentiation marker. Immunol Today.

[58] Kummer JA (2007). Inflammasome components NALP 1 and 3 show distinct but separate expression profiles in human tissues suggesting a site-specific role in the inflammatory response. J Histochem Cytochem.

[59] Qadri M (2018). Recombinant human proteoglycan-4 reduces phagocytosis of urate crystals and downstream nuclear factor kappa B and inflammasome activation and production of cytokines and chemokines in human and murine macrophages. Arthritis Res Ther.

[60] Terkeltaub R, Martin J, Curtiss LK, Ginsberg MH (1986). Apolipoprotein B mediates the capacity of low density lipoprotein to suppress neutrophil stimulation by particulates. J Biol Chem.

[61] Itahana Y (2015). The uric acid transporter SLC2A9 is a direct target gene of the tumor suppressor p53 contributing to antioxidant defense. Oncogene.

[62] Gournas C, Amillis S, Vlanti A, Diallinas G (2010). Transport-dependent endocytosis and turnover of a uric acid-xanthine permease. Mol Microbiol.

[63] Mandal AK, Mercado A, Foster A, Zandi-Nejad K, Mount DB (2017). Uricosuric targets of tranilast. Pharmacol Res Perspect.

[64] Naftalin, R. J. The effects of probenecid and salicylate on uric acid flux across red cell membranes. *J Physiol* **211**, Suppl:47P+ (1970). 5501039

[65] An LL (2014). Complement C5a potentiates uric acid crystal-induced IL-1beta production. Eur J Immunol.

[66] DeForge LE, Remick DG (1991). Kinetics of TNF, IL-6, and IL-8 gene expression in LPS-stimulated human whole blood. Biochem Biophys Res Commun.

[67] Neote K, Darbonne W, Ogez J, Horuk R, Schall TJ (1993). Identification of a promiscuous inflammatory peptide receptor on the surface of red blood cells. J Biol Chem.

[68] Mollnes TE (2002). Essential role of the C5a receptor in E coli-induced oxidative burst and phagocytosis revealed by a novel lepirudin-based human whole blood model of inflammation. Blood.

[69] Angele MK (1999). Sex steroids regulate pro- and anti-inflammatory cytokine release by macrophages after trauma-hemorrhage. Am J Physiol.

[70] Ikejima K (1998). Estrogen increases sensitivity of hepatic Kupffer cells to endotoxin. Am J Physiol.

[71] Sikora J, Mielczarek-Palacz A, Kondera-Anasz Z, Strzelczyk J (2015). Peripheral blood proinflammatory response in women during menstrual cycle and endometriosis. Cytokine.

[72] Hare JM (2008). Impact of oxypurinol in patients with symptomatic heart failure. Results of the OPT-CHF study. J Am Coll Cardiol.

[73] Pfaffl MW (2001). A new mathematical model for relative quantification in real-time RT-PCR. Nucleic Acids Res.

[74] Friedman, M. H. *Principles and Models of Biological Transport*. Second edition edn, (Springer, New York, NY, 2008).

[75] Munn AL (2001). Molecular requirements for the internalisation step of endocytosis: insights from yeast. Biochim Biophys Acta.

[76] Ludden TM, Beal SL, Sheiner LB (1994). Comparison of the Akaike Information Criterion, the Schwarz criterion and the F test as guides to model selection. J Pharmacokinet Biopharm.

